# Brainstem Disconnection Syndrome in a Patient With Respiratory Failure and Failed Weaning From Oxygen: A Case Report

**DOI:** 10.1155/crra/9939709

**Published:** 2026-09-01

**Authors:** Afnan Alshehri, Marah Ashi, Sarah Sulaiman, Ahmed Alzahrani

**Affiliations:** ^1^ Department of Medical Imaging, King Abdulaziz Medical City, Riyadh, Saudi Arabia, ngha.med.sa

**Keywords:** brainstem disconnection, congenital hindbrain malformation, MRI neonate

## Abstract

**Background:**

Brainstem disconnection syndrome (BDS) is an exceedingly rare congenital malformation of the hindbrain characterized by disconnection of the brainstem, often presenting with severe neurological deficits and early neonatal mortality. Whereas previous cases have documented isolated neurological anomalies, this report delineates a distinctive presentation of BDS associated with a specific constellation of multisystem vascular and extracerebral skeletal malformations.

**Case Presentation:**

A 4‐month‐old female infant, born from a discordant twin pregnancy, exhibited persistent neonatal respiratory failure, failure to wean from oxygen therapy, global tonic–clonic seizures, and severe hypotonia. Magnetic resonance imaging (MRI) and magnetic resonance angiography (MRA) of the brain demonstrated a complete absence of the pons, a slender tissue cord connecting the midbrain and medulla oblongata, extensive cerebellar hypoplasia, and nonopacification or aplasia of the basilar and distal vertebral arteries. Whole exome sequencing did not reveal any standard inherited neurodevelopmental mutations; however, genomic analysis identified carrier status for a biologically plausible frameshift variant in TMEM67, a gene heavily implicated in ciliary hindbrain morphogenesis. The implementation of targeted supportive care allowed the patient to survive beyond the typical early neonatal period, with the patient remaining alive at a 12‐month follow‐up.

**Conclusion:**

This case broadens the known phenotypic and radiological range of BDS by reporting new multilevel skeletal and vascular co‐occurring conditions. The notable phenotypic differences seen in this twin pregnancy support the theory of early embryonic vascular disruption. Additionally, the detection of the TMEM67 variant indicates that ciliary gene mutations could be important genetic factors or modifiers in BDS.

## 1. Introduction

Brainstem disconnection syndrome (BDS) is a rare congenital hindbrain malformation characterized by interruption of brainstem continuity, frequently involving the pons, medulla, cerebellar peduncles, and cerebellum. The underlying pathogenesis remains incompletely understood and likely reflects disturbances in early hindbrain patterning, neuronal migration, axonal guidance, or vascular development during embryogenesis. Although several genetic disorders have been associated with posterior fossa and brainstem malformations, a definitive genetic cause has not been established for many cases of BDS [1–5]. Few cases are reported in the literature; most of them have died by the 6th–7th week of age [1, 2].

Brainstem disconnection is generally unrecognized during the prenatal period, although cerebellar hypoplasia can be seen on prenatal US. Neonatal clinical findings vary but typically involve dense neurologic dysfunction [1].

We provide MRI and magnetic resonance angiography (MRA) images illustrating brainstem disconnection, including associated neurological, vascular anomalies, and extracerebral malformations. Additionally, we discuss the condition′s possible causes and differential diagnoses [1].

## 2. Case Presentation

A 4‐month‐old female infant, part of a twin birth, was delivered at full term at 37 weeks via elective cesarean section. She has been a known case of neonatal intensive care unit (NICU) admission since birth, diagnosed with respiratory distress syndrome requiring mechanical ventilation, complicated by difficult intubation and failure to wean from oxygen. The patient has also experienced global tonic–clonic seizures while on phenobarbital and presents with hypotonia. Additionally, she exhibits swallowing difficulties managed with a gastrostomy tube, without fundoplication. The patient was referred to our facility and admitted to the Pediatric Intensive Care Unit (PICU) due to a fever reaching 40°C.

The patient has a history of abnormal movements dating from 1 month of age. The EEG demonstrates a normal background activity with no epileptiform discharges. The patient has received only the birth vaccination.

The vital signs are within normal limits except for documented episodes of tachycardia.

Upon physical examination, the patient appears well‐hydrated, exhibits mild mottled skin, and tolerates oral intake. The patient has severe cerebral palsy affecting both upper and lower limbs.

The patient underwent a bone scan, which revealed questionable osteoblastic activity suggestive of osteomyelitis. The patient was subsequently treated with intravenous antibiotics for 1 month, and the septic workup yielded negative results.

The patient underwent a brain MRI at the referral hospital and was diagnosed with pontocerebellar hypoplasia (PCH), with no report provided.

The patient underwent an upper gastrointestinal examination and a milk scan, which provided evidence of gastroesophageal reflux and delayed gastric emptying observed on the milk scan.

**Figure 1:**
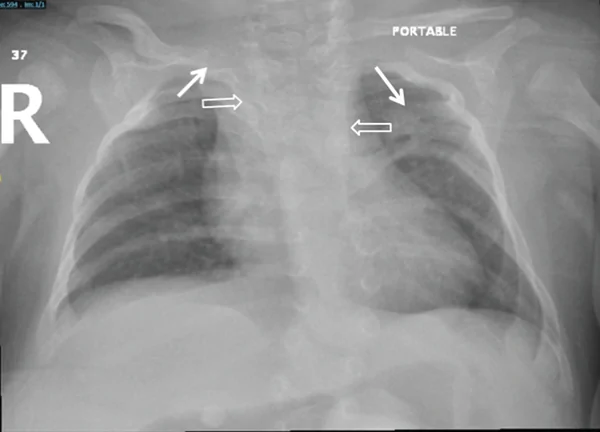
Anteroposterior view of the chest radiograph, demonstrating clear lungs without pleural effusion or pneumothorax and the presence of a normal cardiothoracic silhouette. Bilateral multilevel rib fusion anomalies (solid arrow) and congenital vertebral segmentation and fusion anomalies (empty arrow) are observed, contributing to scoliosis.

During the admission process, the patient underwent a chest x‐ray, which disclosed multiple levels of congenital vertebral segmentation and fusion anomalies along the thoracic spine, as well as several levels of bilateral rib fusion anomalies (Figure 1).

**Figure 2:**
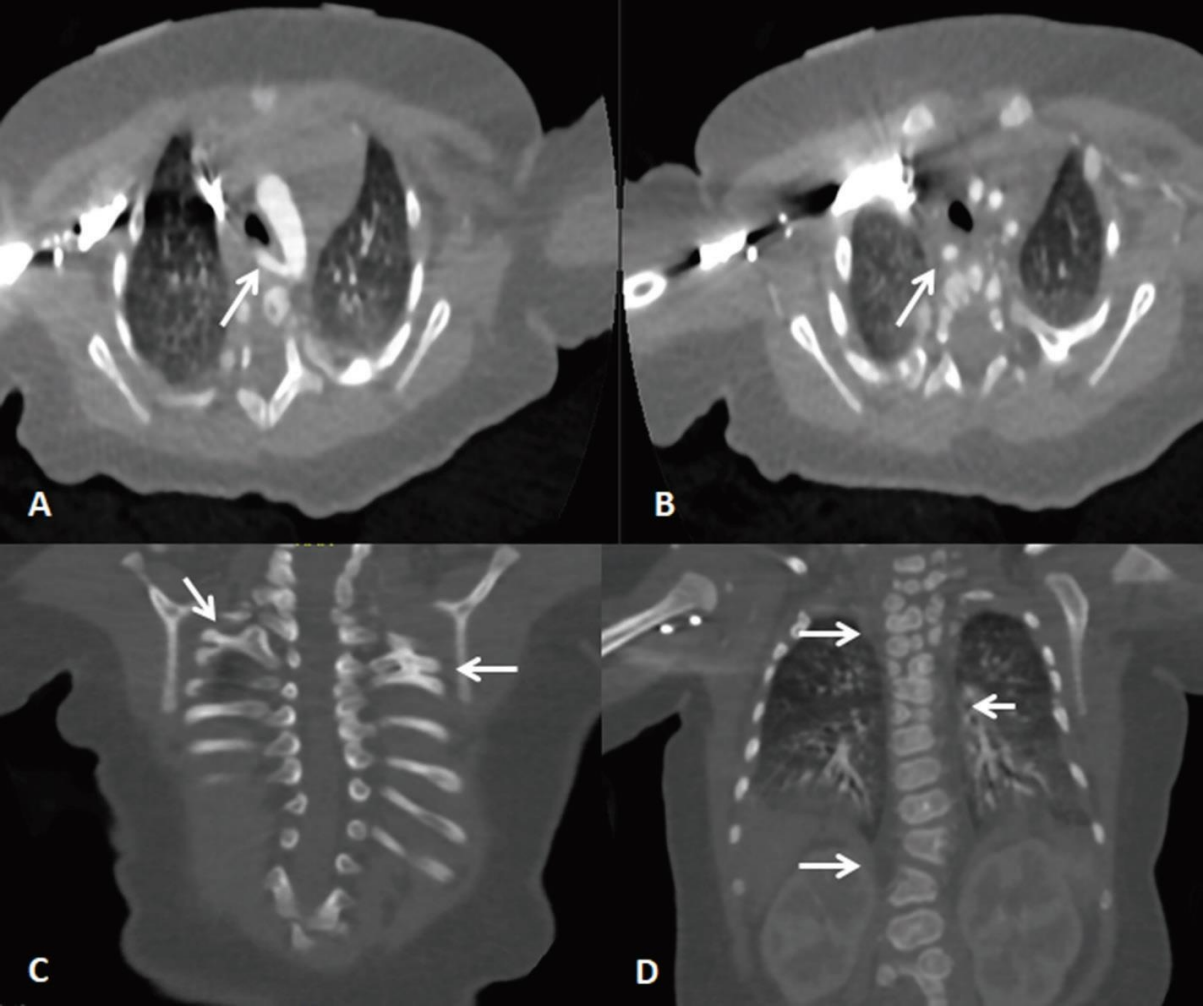
Enhanced chest computed tomography (CT) images. (A,B) Axial view illustrating a right aberrant subclavian artery without airway compression (solid arrows). (C) Coronal image of the bone window revealing bilateral rib fusion anomalies (solid arrows). (D) Coronal view of the bone window demonstrating multilevel congenital segmentation and fusion anomalies (solid arrows).

Several trials to wean the patient from oxygen therapy were unsuccessful, prompting further assessment with chest CT scans to exclude the possibility of airway compression. The chest CT revealed an aberrant right subclavian artery originating distal to the major branches of the aortic arch (namely, the right common carotid, left common carotid, and left subclavian arteries), with no indications of airway compression (Figure 2).

Following 1 month of intravenous antibiotic therapy, the patient underwent a dorsal spine MRI to assess the response to treatment, and a brain MRI was scheduled to document the previously stated diagnosis from the referring hospital. The patient experienced difficulty during the MRI procedure due to challenging intubation, and mild intravenous sedation was administered to facilitate the examination.

MRI data were acquired using a 1.5 Tesla GE Medical System (model Optima MR450w). The standard departmental brain MRI protocol was performed, which included multiplanar T1‐weighted images (T1WIs), T2‐weighted images (T2WIs), diffusion‐weighted images (DWIs), susceptibility‐weighted images (SWIs), and axial 3D time‐of‐flight (TOF) sequences. Briefly, T1WI images were obtained with the following parameters: a field of view (FOV) of 256 × 256 × 160 mm, a flip angle (FA) of 12°, a repetition time (TR)/echo time (TE) of 7.2/2.7 ms, a slice thickness of 1 mm, and an interslice gap of 1 mm. Echo‐planar imaging (EPI) was utilized with a TR/TE of 6427/132.6 ms and an FA of 160°.

Regrettably, the MRI study was constrained, and intravenous gadolinium contrast was not administered.

**Figure 3:**
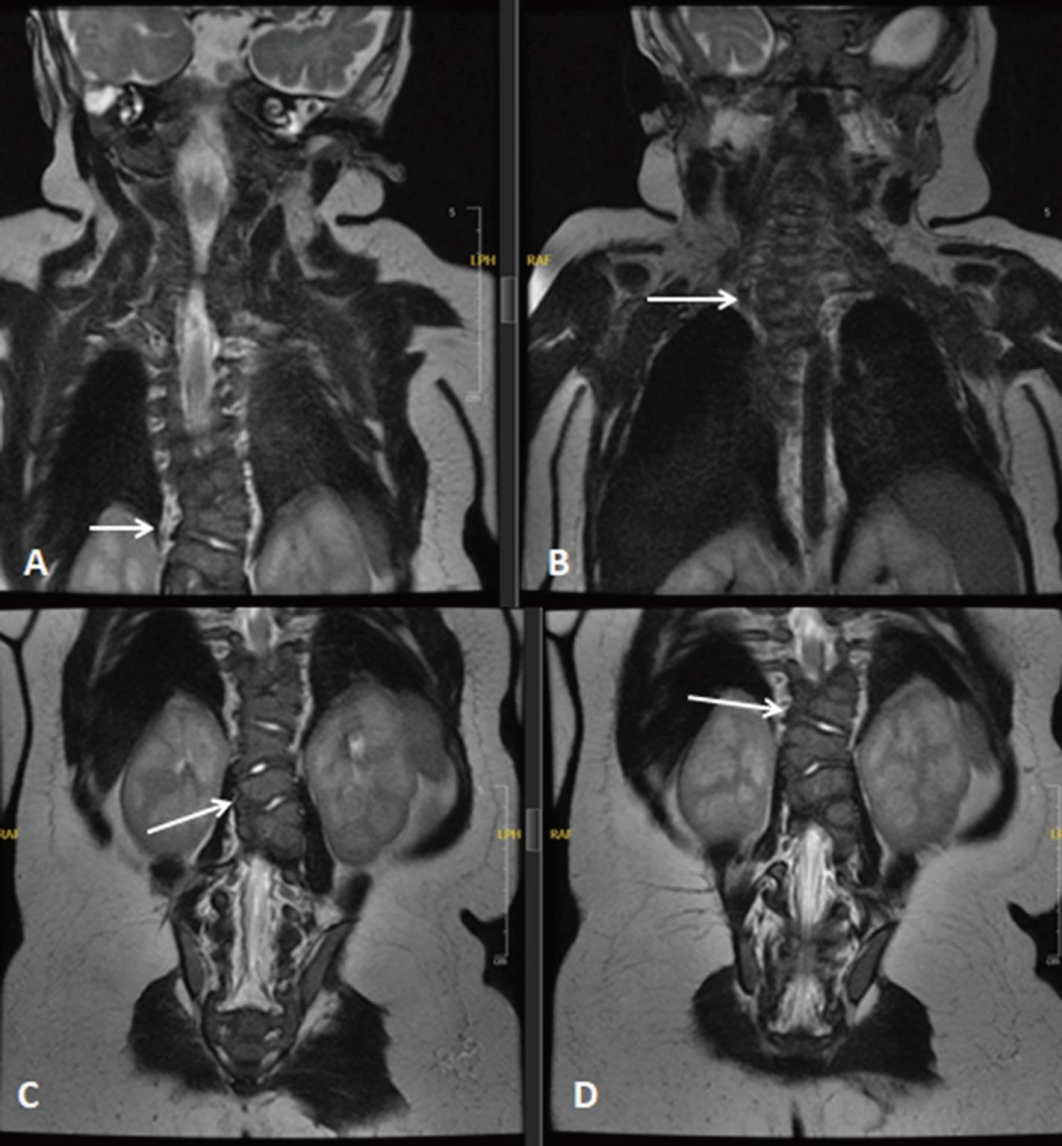
Magnetic resonance imaging (MRI) of the thoracolumbar spine. (A–D) Coronal T2‐weighted images illustrating multiple levels of congenital segmentation and fusion anomalies (solid arrows).

The thoracolumbar spine MRI shows no evidence of osteomyelitis, with redemonstration of multilevel congenital vertebral segmentation and fusion anomalies causing scoliosis (Figure 3).

**Figure 4:**
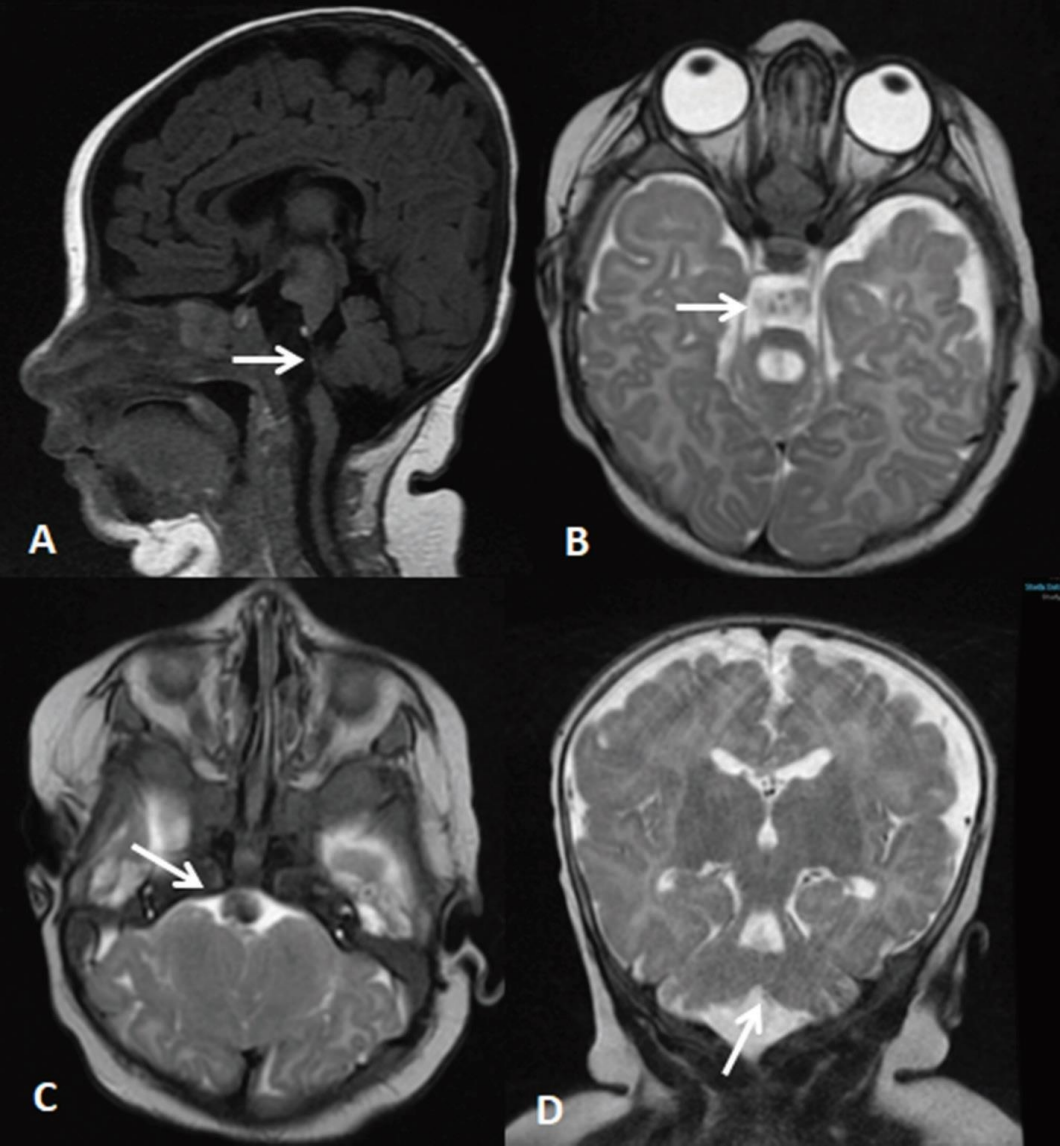
Magnetic resonance imaging (MRI) of the brain. (A) Sagittal T1‐weighted image demonstrates the absence of the pons, with a thin cord connecting the medulla oblongata, midbrain, and cerebellum (solid arrow). (B,C) Axial T2‐weighted images demonstrate the absence of the normal flow void of the basilar artery (solid arrows), suggesting possible aplasia or hypoplasia, and (D) coronal T2‐weighted image reveals hypoplastic cerebellar vermis and hemispheres (solid arrows), along with a prominent cisterna magna and distortion of the fourth ventricle.

The brain MRI reveals a discontinuity between the midbrain and medulla oblongata, with absence of the pons. Instead, a slender cord connects the midbrain to the medulla and cerebellum. Widespread cerebellar hypoplasia is evident, characterized by the absence of both inferior and middle cerebellar peduncles as well as the inferior cerebellar vermis. The fourth ventricle is displaced, and the cisterna magna appears enlarged. Moreover, T2WI of the prepontine cistern demonstrates no signal void of the basilar artery, suggesting possible aplasia or hypoplasia. An additional assessment was conducted using head MRA TOF (Figure 4).

**Figure 5:**
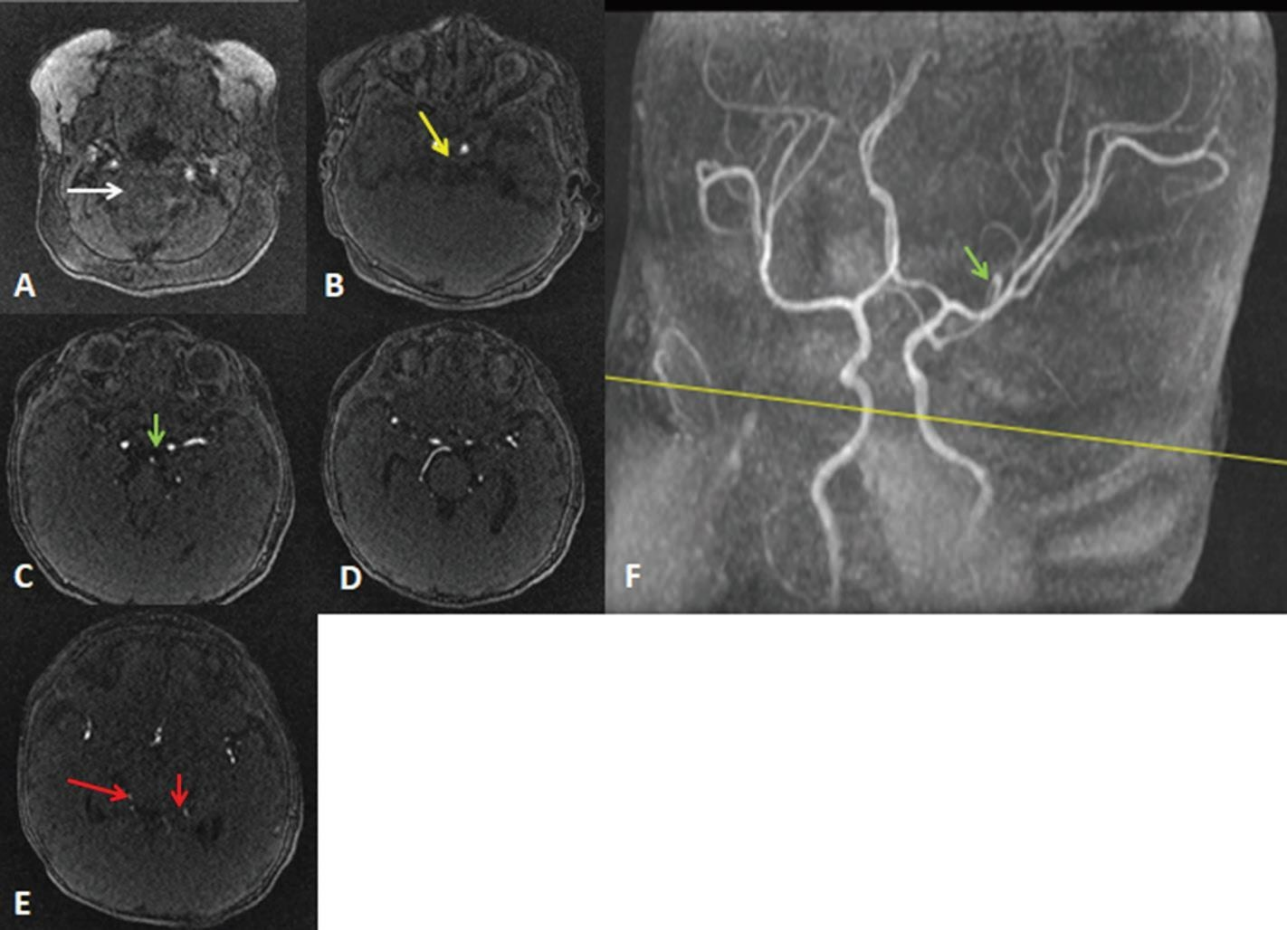
Magnetic resonance angiography of the head utilizing the time‐of‐flight (TOF) technique. (A,B) Axial T1 3D TOF images demonstrate a nonopacified basilar artery (yellow arrow) and the imaged portion of the bilateral distal parts of the vertebral arteries (white arrows). (C,D) The left posterior communicating artery supplying the posterior circulation is indicated (green arrow). (E) Axial T1 3D TOF images reveal bilateral posterior cerebral arteries (red arrows) supplied by the left posterior communicating artery. (F) A subtracted maximum intensity projection (MIP) illustrates that the anterior circulation, including both the anterior cerebral and middle cerebral arteries, as well as both intracranial segments of the internal carotid arteries, is patent, with a hypoplastic or aplastic right posterior communicating artery.

The head MRA (TOF) reveals nonopacification of the basilar and intracranial segments of both vertebral arteries , indicating aplasia or hypoplasia. The intracranial segments of both internal carotid arteries and the anterior circulation are patent, with evidence of the left posterior communicating artery supplying the posterior circulation via bilateral posterior cerebral arteries. There is hypoplasia or aplasia of the right posterior communicating artery (Figure 5).

The advanced MRI studies, such as diffusion tensor imaging (DTI), could not be performed due to the patient′s condition, as the patient experienced difficult intubation.

Genetic counseling was initiated, and additional family history revealed that the parents and two older siblings are healthy. The patient′s twin sister was also healthy. There was no family history of underlying neurodevelopmental abnormalities. Whole exome sequencing (WES) performed at an external facility appeared to be negative. Whole genome sequencing (WGS) testing was also negative; however, carriership findings included CDK10, CLN6, HPS1, PRF1, and TMEM67.

Both the paternal and maternal genetic testing revealed no shared genes.

The patient remains alive to date and has been followed up, reaching 1 year of age.

## 3. Discussion

Recently, significant advancements in the understanding of midbrain and hindbrain (MBHB) malformations have been achieved through the utilization of cutting‐edge genetic and neuroimaging technologies.

The integration of contemporary imaging methodologies with advanced genetic techniques has furnished unprecedented clarity regarding the broad spectrum of developmental disorders impacting the MBHB. Many of these disorders stem from hereditary mutations, whereas a significant proportion are attributable to de novo mutations in the affected individuals and are not inherited.

PCH constitutes a heterogeneous group of disorders characterized by a diminished volume of the pons and cerebellum, exhibiting a range of clinical severities and diverse genetic etiologies. It can be distinguished through imaging findings and associated clinical features. The discovery of causative genes has markedly enhanced the understanding of the disease spectrum and has elucidated critical molecular pathways involved in hindbrain development. Approximately 17 subtypes of PCH exist, each resulting from variants in different genes implicated in transfer RNA (tRNA) splicing (50%), the exosome complex (20%), and tRNA charging (16%). These genetic variations affect RNA processing, protein translation, and mitochondrial regulatory functions, as summarized in Table 1 [6–8].

**Table 1:** Summary of the pontocerebellar hypoplasia type/subtypes and the causative gene.

PCH type/subtypes: Causative gene
PCH1A is due to variants in VRK1
PCH1B is due to variants in EXOSC3
PCH1C is due to variants in EXOSC8
PCH1D is due to variants in EXOSC9
PCH1E is due to variants in SLC25A46
PCH1F is due to variants in EXOSC1
PCH2A is due to variants in TSEN54
PCH2B is due to variants in TSEN2
PCH2C is due to variants in TSEN34
PCH2D is due to variants in SEPSECS
PCH2E is due to variants in VPS53
PCH2F is due to variants in TSEN15
PCH3 is due to variants in PCLO
PCH6 is due to variants in RARS2
PCH7 is due to variants in TOE1
PCH8 is due to variants in CHMP1A
PCH9 is due to variants in AMPD2
PCH10 is due to variants in CLP1
PCH11 is due to variants in TBC1D23
PCH12 is due to variants in COASY
PCH13 is due to variants in VPS51
PCH14 is due to variants in PPIL1
PCH15 is due to variants in CDC40
PCH16 is due to variants in MINPP1
PCH17 is due to variants in PRDM13

Abbreviations: AMPD2, adenosine monophosphate Deaminase 2; CDC40, cell division Cycle 40; CHMP1A, charged multivesicular body protein 1A; CLP1, CLP1 cleavage factor homolog; COASY, coenzyme A synthase; EXOSC1, exosome Component 1; EXOSC3, exosome Component 3; EXOSC8, exosome Component 8; EXOSC9, exosome Component 9; MINPP1, multiple inositol polyphosphate Phosphatase 1; PCH, pontocerebellar hypoplasia; PCLO, piccolo presynaptic cytomatrix protein; PPIL1, peptidylprolyl isomerase Like 1; PRDM13, PR domain zinc finger Protein 13; RARS2, arginyl‐tRNA Synthetase 2; SEPSECS, O‐phosphoserine tRNA:Sec tRNA synthase; SLC25A46, solute carrier Family 25 Member 46; TBC1D23, TBC1 domain family Member 23; TOE1, target of EGR1 scaffold protein domain Containing 1; TSEN2, tRNA splicing endonuclease Subunit 2; TSEN15, tRNA splicing endonuclease Subunit 15; TSEN34 (PCH2C), tRNA splicing endonuclease Subunit 34; TSEN54, tRNA splicing endonuclease Subunit 54; VPS51, vacuolar protein Sorting 51 homolog; VPS53, vacuolar protein Sorting 53 homolog; VRK1, vaccinia‐related Kinase 1.

The mutations in EXOSC3 and VRK1 are correlated with PCH Type 1, which is characterized by the concurrent presentation of spinal muscular atrophy and significant motor weakness. Mutations in the RARS2 gene have been identified in a single case exhibiting a PCH1 phenotype. Unlike PCH Types 2, 4, and 5—primarily associated with mutations in genes encoding tRNA splicing endonucleases, such as TSEN54, TSEN34, and TSEN2—these disorders constitute a clinical continuum. PCH Type 2 represents a relatively milder phenotype, characterized by developmental delay, feeding difficulties, and progressive neurological deterioration, whereas PCH Type 4 presents with severe prenatal manifestations, congenital microcephaly, respiratory failure, and early mortality. A distinctive neuroimaging feature of TSEN‐related PCH is the predominant involvement of the cerebellar hemispheres over the vermis.

Several disorders can clinically and radiologically mimic classical PCH. The calcium/calmodulin‐dependent serine protein kinase (CASK)–related disease and chromatin‐modifying Protein 1A (CHMP1A)–associated PCH share features such as progressive microcephaly, developmental delay, and cerebellar hypoplasia, emphasizing the importance of genetic testing for accurate diagnosis. Similarly, pontine tegmental cap dysplasia (PTCD) is characterized by brainstem abnormalities and multiple cranial nerve deficits, with no causative gene yet identified. The absence of familial recurrence in PTCD suggests a potentially distinct pathogenic mechanism compared with inherited forms of PCH. Furthermore, metabolic disorders such as congenital disorder of glycosylation Type Ia (CDG‐Ia) demonstrate that defects in protein glycosylation can also impair hindbrain development. The broad systemic manifestations observed in CDG‐Ia indicate that cerebellar and pontine hypoplasia may arise as part of a multisystem disorder rather than an isolated neurological condition [6, 9, 10].

In our case, the radiological manifestations do not conform to the classical presentation of PCH or its imitators, and comprehensive genetic investigations have not identified a causative gene mutation in PCH, although numerous forms of PCH are now associated with recognized genetic defects.

An additional distinctive aspect of this case is the existence of a clinically unaffected cotwin exhibiting normal neurodevelopment and devoid of clinicoradiological abnormalities. The discordance between the twins is notable; however, it establishes a potential etiological mechanism for the observed malformations, such as fetal disruptive injuries, which can arise from both maternal and fetal factors that hinder normal fetal development, especially through vascular or circulatory disturbances. Maternal factors encompass systemic hypotension, placental thrombosis, vitamin K deficiency, and drug exposure. Fetal factors include prematurity, circulatory or metabolic failure, twin‐to‐twin transfusion syndrome, cotwin demise, and vascular abnormalities such as those observed in PHACE syndrome. These factors have the potential to disrupt brain development by impairing perfusion, inducing stroke, or causing other vascular insults during critical periods of fetal growth.

The unaffected cotwin expands the phenotypic spectrum of these rare disorders and emphasizes the necessity for additional research to better understand their developmental origins.

Therefore, the lack of classical radiological manifestations of PCH subtypes or their mimics, combined with negative genetic testing for these malformations, may imply BDS.

BDS is a rare congenital malformation of the hindbrain characterized by disruption of brainstem continuity, often involving the pons, medulla, cerebellar peduncles, and cerebellum. The precise pathogenesis remains inadequately understood and likely involves disturbances in early hindbrain patterning, neuronal migration, axonal guidance, or vascular development during embryogenesis. Whereas several genetic disorders have been linked with posterior fossa and brainstem malformations, a definitive genetic etiology has not been confirmed in many cases of BDS [1, 2].

In the case we have presented, the findings may corroborate the vascular disruption hypothesis as a potential mechanism occurring in early pregnancy, which is causally associated with the observed BDS, with the presence of fetal factors related to twin pregnancy. A prior novel case report examined the impact of severe maternal dehydration on the vascular disruption defect hypothesis during the prenatal period, as documented by Vekemans et al. [11].

However, our patient did not have a documented history of repeated maternal admissions attributable to etiologies resulting in severe maternal dehydration, such as severe hyperemesis gravidarum or severe episodes of diarrhea caused by gastrointestinal or upper respiratory tract infections.

An extracranial anomaly is less common [2]. In our case, we document multiple levels of congenital vertebral segmentation and fusion anomalies, scoliosis, rib fusion anomalies, and an aberrant right subclavian artery. In the literature, two documented cases of associated cardiac defects and one case of ectopic thyroid and parathyroid tissue associated with a small thymus have been reported [2].

All cases were full‐term, with polyhydramnios documented on antenatal imaging in some. Antenatal imaging, including ultrasound and fetal MRI, has occasionally described cerebellar hypoplasia and pontine hypoplasia but has failed to detect the brainstem disconnection. Some cases show middle ear abnormalities and a hypothalamic hamartoma, but these were not present in our case [1].

The genetic basis of BDS remains poorly understood, although disturbances in embryonic hindbrain patterning, axonal tract development, neuronal migration, and ciliogenesis have been proposed as pathogenic mechanisms.

Two cases documented in the literature may result from a lack of the EN2 gene [1]. However, no definitive genetic link has been established. Other genes involved in the development of the central nervous and vascular systems, such as FLNA and EZH2, have also been explored. Nevertheless, their role in brainstem disconnection remains under investigation. In our case, the patient exhibits carrier status for several genes, including CDK10, CLN6, HPS1, PRF1, and TMEM67.

Among the identified variants in our patient, the TMEM67 frameshift variant (c.579_580del; p.Gly195IlefsTer13) is of particular interest. TMEM67 encodes the ciliary protein Meckelin and is implicated in ciliopathies such as Joubert syndrome and Meckel syndrome, disorders characterized by cerebellar vermis hypoplasia, abnormal superior cerebellar peduncles, and brainstem malformations. Although BDS has not been definitively linked to TMEM67, the established role of TMEM67 in hindbrain morphogenesis and cerebellar–brainstem development renders this variant a biologically plausible contributor to the observed phenotype.

The CDK10 variant constitutes an early truncating allele, which may hold significance given that CDK10 has been implicated in developmental signaling pathways and ciliogenesis. However, direct correlations between CDK10 deficiency and severe brainstem malformations have not yet been established. The CLN6 variant affects a gene associated with neuronal ceroid lipofuscinosis, a neurodegenerative disorder characterized by progressive cerebellar involvement rather than congenital malformations of the hindbrain. Likewise, the variants in PRF1 and HPS1 are linked to immune dysregulation and lysosome‐related organelle dysfunction, respectively, and presently lack a recognized association with congenital brainstem disconnection.

Previously reported similar cases are summarized in Table 2.

**Table 2:** Summary of previously reported cases considering brainstem pathomorphology, associated neurovascular pathology, and extracerebral manifestations.

Authors (ref)	Patient survival	Brainstem pathomorphology	Associated neurovascular pathology	Extracerebral anomalies
Mamourian and Miller [5]	Died at 6 weeks	Disconnection between pons and medulla, absence of the pons	Cerebellar hypoplasia, no flow void of basilar artery and distal vertebral arteries	None
Saint‐Martin et al. [12]	Not reported	Disruption between mesencephalon and thin medulla, absence of the pons	Unilateral and bilateral nerve palsies—Mobius sequence, cerebellar hypoplasia, poorly defined vermis fissures, small basilar artery	None
Sarnat et al. [13]	Died at 11 days	Absence of the midbrain and upper pons; the nerve‐like cord connects the diencephalons with the caudal brainstem	Global cerebellar hypoplasia, anomalies of the basilar artery and posterior cerebral arteries	Dysmorphic features, micrognathia, low‐set ears, nipples, vertebral and rib abnormalities, hydronephrosis, thyroid ectopia, small thymus, ectopic parathyroid gland, heart defect: Patent foramen ovale (PFO), small ventricular septal defect (VSD)
Sarnat et al. [13]	Died at 5 days	Reduction of the midbrain and upper pons to a thin midline cord	Global cerebellar hypoplasia	None
Bednarek et al. [14]	Died at 11 days	Disconnection between the midbrain and thin pons	Cerebellar and vermian hypoplasia, absence of the right superior cerebral peduncle, hypoplastic left one	None
McCann et al. [4]	Died in early neonate	Disconnection between pons and medulla	Cerebellar and vermian hypoplasia, hypoplastic optic nerves	None
Poretti et al. [3]	Died at 3 weeks	Pontomedullary disconnection	Cerebellar hypoplasia, absence of basilar artery	Heart defect: Transposition of great vessels (TGA), pulmonary atresia (PA), ventricular septal defect (VSD)
Barth et al. [15]	Died at 5 days	Transection and loss of the major part of the pons and medulla oblongata (absence of the midpontine level of the pons); fragments of tissue connect pons and medulla oblongata, syrinx of the brainstem	Cerebellar and vermian hypoplasia, extremely thin (in autopsy) basilar artery	None
Jurkiewicz et al. [2]	Alive at 5 months	Disruption between midbrain and medulla, absence of the pons, thin cords connect the midbrain with medulla oblongata and midbrain with cerebellar hemispheres	Hypothalamic hamartoma, cerebellar and vermis hypoplasia, no flow void of basilar artery and distal vertebral arteries, enlargement of the cisterna magna, dilated lateral and III ventricles	Dysmorphic features, vertebral/rib anomalies, scoliosis, micrognathia, middle ear defects, and short trachea
Alshehri et al. (current)	Alive at 12 months	Disruption between midbrain and medulla oblongata with absence of pons, presence of thin cords connecting the midbrain to medulla oblongata and midbrain to both cerebellar hemispheres	Absence of inferior cerebellar vermis with malposition of the fourth ventricle and prominent cisterna magna. Aplasia/hypoplasia of basilar/vertebral arteries; patent left PCoA supplying posterior circulation	Vertebral segmentation and fusion anomaly, rib fusion anomaly, and scoliosis. Aberrant right subclavian artery

In conclusion, TMEM67 emerges as the most compelling candidate among the detected variants based on current knowledge of cerebellar and brainstem development. Nevertheless, because BDS is genetically heterogeneous and incompletely characterized, a contributory role of additional variants or oligogenic mechanisms cannot be excluded. Further genetic studies, segregation analysis, and identification of similar cases will be required to clarify the pathogenic relationship between these variants and the brainstem disconnection phenotype.

## 4. Conclusion

Disconnection of the brainstem is an exceedingly rare and still inadequately understood condition in neonates. Further studies are requisite to elucidate the potential etiology of this malformation. This hindbrain abnormality may be associated with extracerebral findings, as documented in our case, including congenital vertebral segmentation and fusion anomalies, scoliosis, and fusion rib anomalies. Additionally, other reported cases in the literature have documented findings such as hypothalamic hamartoma, ectopic thyroid and parathyroid glands, a small thymus, hydronephrosis, dysmorphic features, and cardiac defects (including VSD, PDA, and TGA).

The identification of TMEM67 is most biologically significant for the observed brainstem and cerebellar abnormalities, as it is known to contribute to ciliopathy‐related hindbrain malformations. Conversely, other variants detected, such as CLN6, PRF1, CDK10, and HPS1, have either indirect or limited links to brainstem development. Although these variants might influence the neurological phenotype, there is currently insufficient evidence to confirm a direct causal link to BDS. Additional research—including segregation analysis, functional studies, and finding similar cases—is needed to determine their pathogenic relevance.

## Funding

No funding was received for this manuscript.

## Ethics Statement

This case report was formally reviewed and approved by the Institutional Review Board (IRB) of the King Abdullah International Medical Research Center (KAIMRC), King Abdulaziz Medical City, Ministry of National Guard Health Affairs, Riyadh, Saudi Arabia (National Committee of Bioethics Registration No: H‐01‐R‐005; IRB Approval Number: 00000230425) on October 1, 2025. Written informed consent for publication was obtained from the parent of the minor patient.

## Conflicts of Interest

The authors declare no conflicts of interest.

## Data Availability

My case report is not available or published in any journal or website.
